# Depressive Symptoms and Category Learning: A Preregistered Conceptual Replication Study

**DOI:** 10.5334/joc.35

**Published:** 2018-06-26

**Authors:** Isa Rutten, Wouter Voorspoels, Sara Steegen, Peter Kuppens, Wolf Vanpaemel

**Affiliations:** 1 University of Leuven, BE

**Keywords:** Categorization, Executive functions, Emotion and cognition

## Abstract

We present a fully preregistered, high-powered conceptual replication of Experiment 1 by Smith, Tracy, and Murray (1993). They observed a cognitive deficit in people with elevated depressive symptoms in a task requiring flexible analytic processing and deliberate hypothesis testing, but no deficit in a task assumed to require more automatic, holistic processing. Specifically, they found that individuals with depressive symptoms showed impaired performance on a criterial-attribute classification task, requiring flexible analysis of the attributes and deliberate hypothesis testing, but not on a family-resemblance classification task, assumed to rely on holistic processing. While deficits in tasks requiring flexible hypothesis testing are commonly observed in people diagnosed with a major depressive disorder, these deficits are much less commonly observed in people with merely elevated depressive symptoms, and therefore Smith et al.’s (1993) finding deserves further scrutiny. We observed no deficit in performance on the criterial-attribute task in people with above average depressive symptoms. Rather, we found a similar difference in performance on the criterial-attribute versus family-resemblance task between people with high and low depressive symptoms. The absence of a deficit in people with elevated depressive symptoms is consistent with previous findings focusing on different tasks.

Smith, Tracy, and Murray (1993) investigated whether depressive symptoms eliminate sophisticated cognitive strategies, but leave performance on tasks requiring simple or more automatic strategies intact. In particular, they presented participants with two category learning tasks, in which they were asked to classify stimuli (four-letter nonsense words) in one of two experimenter-defined categories. Crucially, the tasks differed in the cognitive processes assumed necessary to successfully classify the stimuli. In the criterial-attribute (CA) task, a diagnostic feature differentiated between the categories whereas in the family-resemblance (FR) task, there was no such criterial feature but all stimulus attributes were equally informative about the discriminating feature of the categories. Smith et al. (1993) assumed that in the CA task successful learning hinged on flexible analytic processing and deliberate hypothesis testing, whereas learning in the FR task was assumed to depend on a more automatic, holistic strategy, in which relying on the global degree of resemblance between the category members was sufficient to learn to differentiate between categories. In two experiments, Smith et al. (1993) compared the performance of participants with high depressive symptoms to participants with low depressive symptoms. The group with high depressive symptoms showed impaired performance on the CA task, which demanded a sophisticated cognitive strategy, but not on the FR task, where a sophisticated strategy was not required.

In one of the experiments (Experiment 2), the participants (*n* = 131) were psychiatric or non-psychiatric in- and outpatients, and they were divided into three groups based on their level of depressive symptoms, as was assessed using the Beck Depression Inventory (BDI; Beck, 1967). The results showed that the group with the highest levels of depressive symptoms (mean BDI score = 33.6) showed impaired performance on the CA task, but not on the FR task. The observed pattern of results makes good sense, as the participants in the higher depressive symptoms groups were more likely to have a psychiatric diagnosis, in particular a major depressive disorder (MDD), and were more likely to be inpatients, instead of outpatients (Smith et al. 1993). This finding is consistent with related empirical work, suggesting that cognitive performance of people suffering from MDD is often found to be impaired on many, but not all kinds of tasks. The extent to which conscious, strategic and effortful cognitive processing is in play, seems to be a crucial factor. Compared to healthy controls, people with MDD generally perform more poorly on tasks relying on elaborative cognitive strategies (in other words, tasks relying on executive functions; see Snyder, 2013 for a review), however, similar impairments are not observed with respect to tasks that rely mainly on automatic cognitive processes and not on effortful cognitive strategies.

In Smith et al.’s (1993) other experiment (Experiment 1) the same experimental and analytical procedure was conducted as in their Experiment 2, but the participants (*n* = 135) were not psychiatric patients, instead they were members of the university community with varying degrees of depressive symptoms. Participants were divided into two groups based on the mean BDI score of the sample (*M* = 10.03): A group with below average depressive symptoms (*M* = 5.6), and a group with above average depressive symptoms (*M* = 17.25). The group with elevated depressive symptoms was again found to show impaired performance on the CA, but not on the FR task. This finding is important because the mean BDI score in the high symptoms group of Experiment 1 (*M* = 17.25) is considerably smaller than the mean BDI score in the high symptoms group of Experiment 2 (*M* = 33.6),^1^ and evidence for cognitive deficits in individuals with elevated depressive symptoms, but without a diagnosed MDD, is mixed (Snyder, 2013). For example, Bunce, Handley, and Gaines (2008) did not observe a deficit in executive functions, measured across different tasks, including a set switching task (involving flexible analysis), in people with elevated depressive symptoms.

In sum, Smith et al. (1993) hypothesized and observed a deficit in flexible analytic processing related to depressive symptoms in both a patient and non-patient sample, but in the light of later evidence, we would not expect a (strong) relationship between depressive symptoms and deficits in flexible analytic processing in a non-patient sample. Therefore, we attempted to replicate Experiment 1 of Smith et al. (1993), using high power, preregistration, and maximal research transparency. Unlike the original study, which relied on the BDI to assess depressive symptoms, we used the Center for Epidemiologic Studies – Depression Scale (CES-D; Radloff, 1977), a widely accepted questionnaire to test for depressive symptoms in the general population. Because of this difference, our study should be considered a conceptual replication (LeBel, McCarthy, Earp, Elson, & Vanpaemel, in press).

## Method

### Transparency

Before data collection, we preregistered a document with an elaborate description of methods and analyses on Open Science Framework (OSF; https://osf.io/rqzm7/). The preregistration document contains a detailed description of the sampling plan and recruitment, experimental procedures, data processing and analyses, and known methodological differences from the original study. This preregistration document was followed exactly, unless otherwise noted. We followed Smith et al. (1993) closely in their experimental and analytical approach, the main difference being the use of the CES-D to assess depressive symptoms instead of the BDI. A description of other, minor differences between both studies can be found in the preregistration document, section *4*. *Known Differences from Original Study*. We report all measures collected for our experiment. The data, R code for analysis, power protocols, and experiment materials are available on: https://osf.io/auzgp/. Throughout, we set the significance level at. 05, unless noted otherwise. All *F*-tests were one-tailed. The ANOVAs were computed using the ez package (Michael, 2013) in R. Bayesian analyses were performed using the BayesFactor package (Morey, & Rouder, 2015) in R. The power computations were performed using G*Power 3 (Faul, Erdfelder, Buchner, & Lang, 2009).

### Participants

After receiving approval of the Ethical Commission of the Faculty of Psychology and Educational Sciences of the University of Leuven (approval code: G- 2014 12 118), a total of 243 students of the Psychology Department participated for one course credit. The stopping rule for data collection was the end of collective research sessions, in which all first-year psychology students were invited to participate. Based on similar sessions organized earlier in the academic year, we expected between 300 and 350 students to participate. In total, 243 students actually participated in our collective research sessions (level of participation in such sessions is known to vary considerably, and decreases steadily throughout the academic year). The participants had a mean age of 19.01 years (*SD* = 2.64; range = 17–41), with 12 years of schooling for the majority of participants (*n* = 192), and more than 15 years of schooling for 51 participants. The majority of participants were women (85%). For five participants, data of the CES-D were missing, resulting in a usable sample of 238 participants on which the analyses were conducted. No other data were excluded.

On the basis of the *F* statistics, and degrees of freedom reported in the original article, we calculated the effect sizes of the interaction effects between group (above and below average) and task (CA–FR), using \eta _p^2 = {\textstyle{{F \times d{f_{effect}}} \over {F \times d{f_{effect}}\, + \,d{f_{error}}}}} (Bakeman, 2005), resulting in \eta _p^2 = 0.05 for the first dependent variable (*total correct*), and \eta _p^2 = 0.04 for the second dependent variable (*blocks-to-learn*). Given these effect sizes and assuming a CA/FR correlation of zero, the sample size of 238 participants corresponded to a power of 0.99 and 0.97 for the dependent variables *total correct* and *blocks-to-learn*, respectively.^2^

### Materials and procedure

After providing demographic information, participants completed the CA and FR task. The stimuli in the tasks were pronounceable, nonsense four-letter words. We followed Smith et al.’s (1993, pp. 333) description of stimulus creation in order to obtain the exact same stimulus set. Tables 1 and 2 show example stimuli of the CA and FR task, respectively. In the CA task, one letter position, the criterial attribute, was indicative for group membership, whereas in the FR task no such criterial attribute was available to identify group membership. Each task consisted of 48 trials (divided into six blocks), with each trial containing one stimulus to be classified in the correct category (I or II). Participants could learn which words belonged to category I and which to category II, by relying on corrective feedback after each classification decision. After completing both tasks, participants answered categorization style and effort questions, and completed the CES-D. A more extended description of the experimental materials and procedure can be found in the preregistration document.

**Table 1:** Example of stimuli in the CA task. The second letter position is indicative for category membership. I refers to category 1 membership, E refers to category II membership.

Category I		Category II	

Structure	Stimuli	Structure	Stimuli

0000	GIRU	1111	LETA
0011	GITA	1100	LERU
1010	LITU	0101	GERA
1001	LIRA	0110	GETU

**Table 2:** Example of stimuli in the FR task. Each word in category I has three letter positions referring to category I (positions corresponding with 0). Each word in category II has three letter positions referring to category II (positions corresponding with 1).

Category I		Category II	

Structure	Stimuli	Structure	Stimuli

1000	LIRU	0111	GETA
0100	GERU	1011	LITA
0010	GITU	1101	LERA
0001	GIRA	1110	LETU

## Results

### Data processing

To determine above average and below average depressive symptoms groups, a split at the CES-D sample mean was applied. A split at the mean (14.60; *SD* = 11.59) resulted in an above average depressive symptoms group of 101 participants (mean CES-D score = 25.61), and a below average depressive symptoms group of 137 participants (mean CES-D score = 6.47). Two dependent variables were created, both adopted from Smith et al. (1993): *Total correct*, reflecting the sum of all trials in which correct responses were given, and *blocks-to-learn*, reflecting the number of the first block in which seven or more correct responses were given. Participants who never obtained seven or more correct responses in one of the six blocks of a task, were given a score of 7 and considered *failures-to-learn* for that task as they never reached this performance criterion.

### Main preregistered analyses

**Figure 1:**
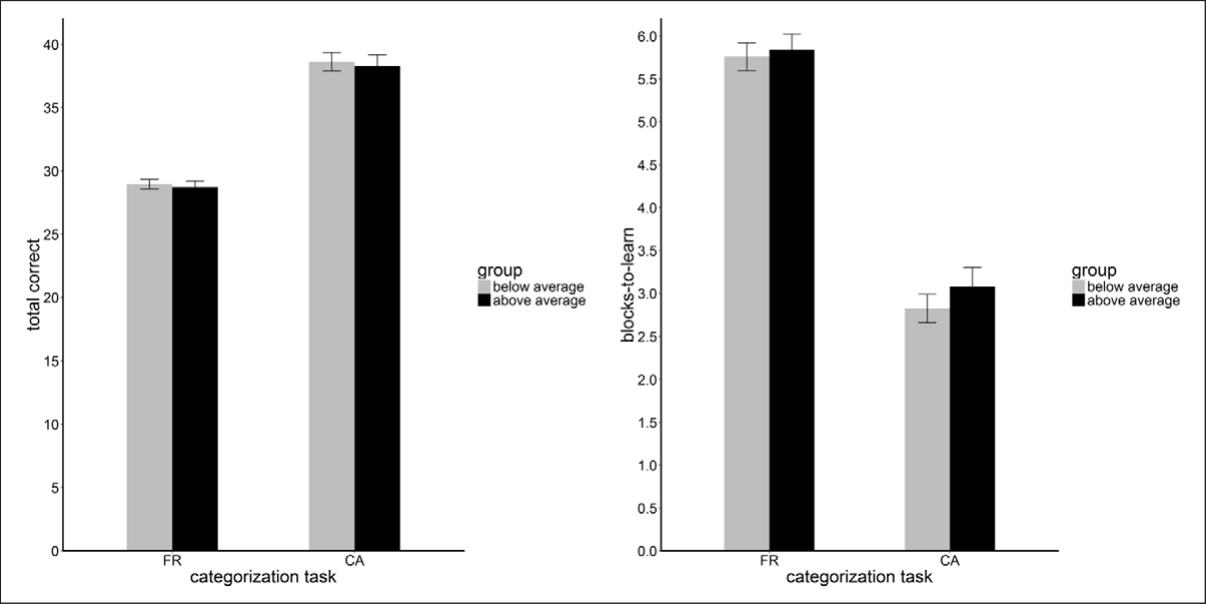
Task Performance. Performance on the family-resemblance (FR) and criterial-attribute (CA) tasks by people with below average and above average depressive symptoms, with error bars showing the standard errors of the sample means.

Figure 1 presents the comparison of above and below average groups’ performance on the CA and FR task for both dependent measures. Each dependent measure was entered into a three-way ANOVA (type II), with task (CA and FR) as a within-subject factor, and group (above average versus below average symptoms) and order (CA-FR versus FR-CA) as two between-subject factors. Both ANOVAs showed evidence for a main effect of task, *F*(1, 234) = 245.62, *p* < 0.001, *MSe* = 44.93, generalized eta squared (\eta _G^2) = 0.34, for *total correct*, and *F*(1, 234) = 248.71, *p* < 0.001, *MSe* = 3.92, \eta _G^2 = 0.36, for *blocks-to-learn*. Participants performed significantly better on the CA task, compared to the FR task. Further, the interaction between order and task was significant, *F*(1, 234) = 10.92, *p* = 0.001, *MSe* = 44.93, \eta _G^2 = 0.02, for *total correct*, and *F*(1, 234) = 12.73, *p* < 0.001, *MSe* = 3.92, \eta _G^2 = 0.03, for *blocks-to-learn*. Participants showed a better performance on the task (CA or FR) that was administered first.

Unlike the original study though, we did not observe evidence for a significant interaction between group and task, *F*(1, 234) = 0.01, *p* = 0.93, *MSe* = 44.93, for *total correct*, and *F*(1, 234) = 0.23, *p* = 0.63, *MSe* = 3.92, for *blocks-to-learn*. Given the absence of significant interaction effects between group and task, the preregistered post-hoc t-tests are redundant, and are not reported. Results of secondary preregistered analyses can be found in the supplementary file.

### Non-preregistered analyses

Additionally, we performed Bayesian analyses, yielding similar results.^3^ The Bayes factors revealed massive evidence for a main effect of task (*BF*_10_ > 1000, for both dependent variables), strong evidence for an order by task interaction (*BF*_10_ > 40, for both dependent variables), and, crucially, substantial evidence for the absence of an interaction between group and task, with *BF*_01_ > 5 for both dependent variables.

We also performed analyses in which an extremer group differentiation was considered, a group with CES-D scores below percentile 33 (<8) versus a group with scores above percentile 66 (>18). These analyses revealed the identical pattern of results: No significant interaction effects between group and task. When the CES-D score was included as a continuous predictor in the model, replacing the categorical group variable, again no significant interaction effect between CES-D score and task was observed. Details of all these non-preregistered analyses can be found in the supplementary file.

## Discussion

Unlike Smith et al. (1993), who found that people with elevated depressive symptoms showed a deficit in sophisticated, flexible analytic processing, but an intact ability of more automatic processing, our data showed that both groups performed better on the CA than the FR task, with a similar difference in performance on the two tasks. Thus, irrespective of the presence of depressive symptoms, people demonstrated a comparable ability to use flexible analytic processing strategies. Bayes factors indicated the data were in favor of the null model, in which no difference between both groups in performance on the CA task was assumed. Also when considering depressive symptoms as a continuous predictor or considering an extremer group differentiation, no significant difference in performance depending on depressive symptoms could be observed.

In further analyses (which can be found in the supplementary file), regarding participants’ self-reported categorization strategies, we found evidence suggesting that the dominant strategy in the FR task was not necessarily purely holistic, but could be at least for some part being influenced by single, non-criterial attribute responding, as suggested by Wills, Inkster, & Milton (2015). Additionally we found that, in general, more effort was invested in the CA task, compared to the FR task, again suggesting single, non-criterial attribute responding in the FR task, as this strategy is thought to be less effortful than overall similarity responding or reliably discovering the criterial attribute (Wills et al., 2015). While outside the scope of the current replication, it is interesting to revisit our findings from the perspective of the underlying categorization style strategies applied in both tasks in further research (Wills et al., 2015).

Our study should be treated as a conceptual replication of Smith et al. (1993). Unlike the original authors, who relied on the BDI to screen for depressive symptoms, we relied on the CES-D. We preferred to use the CES-D over the BDI because the CES-D has been found to be better at detecting small individual differences in severity of depressive symptoms in both student and outpatient samples, compared to the BDI (Santor, Zuroff, Ramsay, Cervantes, & Palacios, 1995). Therefore it is not unlikely that these two questionnaires tap into slightly different aspects of depression, which is supported by observed correlations between the CES-D and the BDI ranging between 0.50 and 0.70 (Hanewald, 1987; Wilcox, Field, Prodromidis, & Scafidi, 1998). Yet, if the use of different depression questionnaires leads to qualitatively different patterns in the results, the implications of our study do not only speak to our replication attempt, but to the whole field of research in depression. Indeed, the results of studies relying on sum scores of different depression questionnaires are often compared without taking the differences between these questionnaires in assessed symptoms into account (Fried & Nesse, 2015). This is illustrated by meta-analyses summarizing findings across different studies, relying on different depression questionnaires (e.g. Hallion, & Ruscio, 2011). Fried and Nesse (2015) recommend considering results robust only if they are observed across different depression scales.

Besides in operationalization of depressive symptoms, our replication study differs from the original one in terms of sample demographics. We do not expect, however, that differences in age (21 in the original vs 19 in the current study), gender (54% female vs 85% female, respectively), nationality (American vs Belgian, respectively) or occupational status (university community members vs students, respectively) should have influenced the results in any major way. If there was indeed a link between elevated depressive symptoms and selective impaired performance, our failure to replicate should have been accompanied by a difference in depressive symptoms between the original and our study. However, the mean score on depressive symptoms in the current sample was very comparable to the mean of Smith et al. (1993). To categorize participants in an above versus below average depressive symptoms group, a split at the sample mean was applied, leading to a cutoff value of 10.03 for the BDI in the original study, and 14.6 for the CES-D in the current study. A score of 10.03 on the BDI falls below the cutoff for moderate depressive symptoms (Beck, 1967). For the CES-D, the cutoff for clinically relevant depressive symptoms is 16 (Smarr & Keefer, 2011), so a score of 14.6 on the CES-D falls within the same severity range as a score of 10.03 on the BDI. This makes it unlikely that a difference in severity of depressive symptoms between both studies lies at the base of our results.

To conclude, the absence of an executive functions-related deficit in people with elevated depressive symptoms is consistent with previous findings focusing on different tasks (e.g. Bunce et al., 2008), and is an important finding as it relates to many people: About 36% of the students had a CES-D score above 17 (above the cutoff of 16, pointing at clinically relevant depressive symptoms; Smarr & Keefer, 2011).

## Additional File

The additional file for this article can be found as follows:

10.5334/joc.35.s1 Supplementary file. Secondary preregistered and exploratory analyses.

## Data Availability

The data are available on OSF: https://osf.io/auzgp/.

## Footnotes

Smith et al. (1993) however warned against a direct comparison of both experiments because of the difference between both samples.

The observed CA/FR correlations were 0 and –0.06 for total correct and for blocks-to-learn respectively.

Bayes factors were calculated by comparing a full model (including all main and interaction terms) with a model without the effect of interest. *BF*_10_ indicates that the BF was calculated with the full model in the numerator, and the restricted model in the denominator, with values greater than 1 indicating evidence for the full model, whereas *BF*_01_ indicates that the BF was calculated with the restricted model in the numerator, and the full model in the denominator, with values greater than 1 indicating evidence for the restricted model. For all reported results, the scale factor in the prior was set to medium, but similar results were obtained with different values for the scale factor.
