# Supplementary material for: Depressive Symptoms and Category Learning: A Preregistered Conceptual Replication Study
Source: J Cogn. 2018 Jun 26;1(1):34. doi: 10.5334/joc.35 (PMC6634606; doi:10.5334/joc.35)
Supplement: Supplementary file. — Secondary preregistered and exploratory analyses. [file joc-1-1-35-s1.pdf]

Supplementary File: Secondary Preregistered and Exploratory Analyses

A. Preregistered analyses: Data cleaning and processing

Across experimental groups and tasks, the two dependent measures; total correct responses and blocks-to-learn, showed a correlation of -0.84 (p < 0.001), comparable with the correlation of -0.78 in the original study (Smith et al., 1993). For the CA task, the two dependent variables showed a correlation of -0.90 (p < 0.001), exactly the same value as in the original study (Smith et al., 1993) but for the FR task, they correlated less strongly (r = -0.52, p < 0.001), with a correlation of -0.61 obtained by Smith et al. (1993).

B. Preregistered analyses: Analyses as in the original study

To investigate the possibility of a restriction of range in the dependent variables, standard deviations were calculated. For the dependent variable *total correct* responses, the standard deviation of the CA task (*SD* = 8.52) almost doubled the standard deviation of the FR task (*SD* = 4.61). For the dependent variable *blocks-to-learn,* the standard deviations were more similar (CA: *SD* = 2.06, FR: *SD* = 1.86). The variability in performance on the FR task seems to be considerably smaller, but as we are mainly interested in the difference between both groups on the CA task, we do not suspect this having influenced our results substantially.

We also examined whether there were differences in terms of *failures-to-learn* the correct classification (participants who never obtained seven or more correct responses in one block) between the above average and below average groups. For the CA task, 21% of the above average group versus only 11% of the below average group were classified as *failures-to-learn*. For the FR task, these percentages were 65% and 64% for the above average and below average groups, respectively. This seems to suggest that the CA task, but not the FR task, was more difficult for people in the above average depressive symptoms group than for people in the below average depressive symptoms group. However, when comparing the performance of the median participant (score on CES-D) of the above average and below average groups, the opposite conclusion could be made: The median participant of the above average group reached criterion before the median participant of the below average group (respectively, after 2.71 blocks versus after 3.00 blocks for the CA task; after 5.29 blocks versus after

6.75 blocks for the FR task). Thus, evidence for performance impairment in the above average group is only weakly present in terms of slightly more *failures-to-learn*, and not present in all the other indicators.

The preregistered analysis plan contained additional analyses for male and female participants separately, to test for a confounding influence of gender in the results. Smith et al. (1993) did not observe a different pattern in results when performing the analyses separately for male and female participants, but we were unable to test this finding given the limited amount of male participants (15%) in the current study.

C. Preregistered analyses: Analyses not part of the original study

Cronbach's alpha was calculated on the three items assessing the invested effort in the FR task and on the three items assessing effort invested in the CA task, resulting in alpha's of 0.81 and 0.79, respectively. The items were combined into a single variable for each task. A two-way mixed ANOVA (type II) with effort as dependent variable, task (CA and FR) as a within-participant factor, and group (above versus below average) as a between-subject factor, showed a main effect of task, $F(1, 236) = 14.30$, $p < 0.001$, $MSe = 2.12$, $\eta^2_G = 0.01$, and a main effect of group, $F(1, 236) = 6.13$, $p = 0.01$, $MSe = 8.56$, $\eta^2_G = 0.02$, but no interaction between task and group, $F(1, 236) = 0.27$, $p = 0.61$, $MSe = 2.12$. Overall, more effort was invested in the CA task (M = 11.83, SD = 2.33), compared to the FR task (M = 11.33, SD = 2.33), and people in the above average group invested less effort in both tasks (M = 11.19, SD = 2.38), compared to people in the below average group (M = 11.86, SD = 2.27), see Figure C1. After including effort as predictor in the main analyses with the performance indices (*total correct* and *blocks-to-learn)* as dependent variables, using a linear mixed effects model (nlme package R; Pinheiro et al., 2013), the initial conclusion remained the same: No significant interaction between group and task could be observed, $t(232) = 0.82$, $p = .41$, for *total correct*, and $t(232) = 0.02$, $p = .98$, for *blocks-to-learn*.

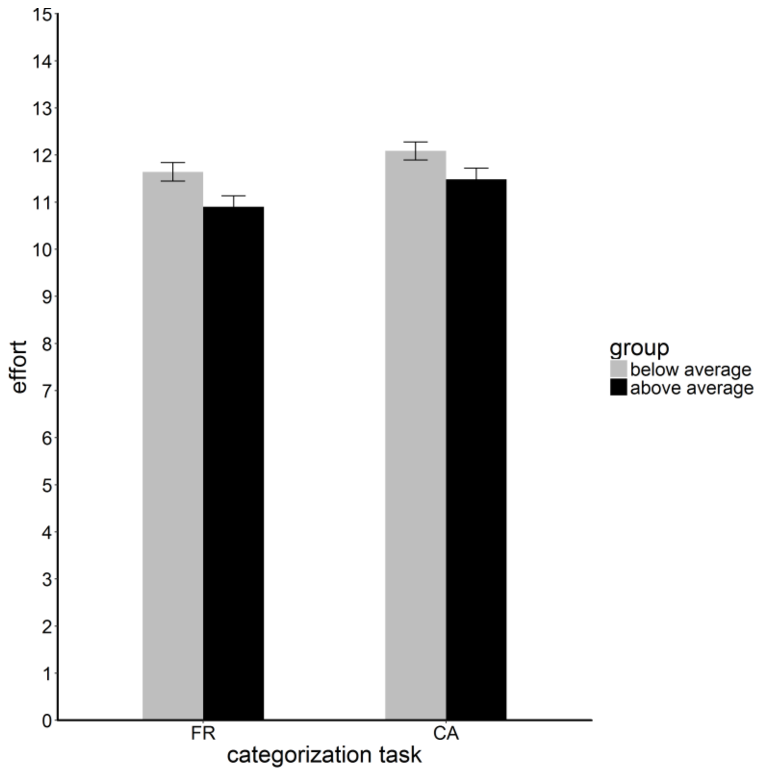

Figure C1. Self-reported amount of effort invested in the FR and CA task by the below and above average depressive symptoms groups, with error bars showing the standard errors of the sample means.

Regarding the self-report categorization style questions, *analytic processing* and *rule-based* (which can be found on https://osf.io/auzgp), two two-way mixed ANOVAs were performed (type II) with task (CA and FR) as a within-participant factor, group (above versus below average) as a between-participant factor, and either *analytic processing* or *rule-based* as dependent variable. Concerning *analytic processing*, the ANOVA showed only a main effect of task, $F(1,236) = 5.80$, $p = 0.02$, $\eta_G^2 = 0.01$. Participants gave a higher score on the *analytic processing* item for the FR task (M = 1.76, SD = 0.86) compared to the CA task (M = 1.57, SD = 1.13). The mean analytic processing scores of 1.76 and 1.57 reflect the use of both a holistic impression of the words, and inspection of the individual letters. For more in-depth analysis of the specific categorization styles used in the two tasks, and in particular to distinguish between overall similarity responding or holistic responding and single, non-criterial, attribute responding in the FR task, one can apply response-set analysis, as presented by Wills, Inkster, & Milton (2015), on our data (https://osf.io/auzgp/).

Concerning *rule-based* as dependent variable, again only a main effect of task was observed, $F(1,236) = 218.26$, p < 0.001, $\eta^2_G = 0.29$. On average, participants had a clearer idea of a rule in the CA task (M = 2.34, SD = 1.01), compared to the FR task (M = 1.14, SD = 0.86), what makes sense given that only in the CA task a clear, correct rule (the criterial attribute) could be detected. More additional analyses, such as calculating a switch index, a single-attribute index, a backward learning curve, were described in the preregistration document with the goal of investigating the precise nature of the difference in performance between the above versus below average depressive symptoms groups. As no significant performance difference was observed between people with above versus below average depressive symptoms, we did not perform all these preregistered additional analyses.

D. Non-preregistered (exploratory) analyses

One explanation for the absent performance difference between both groups is that the groups, which were constructed using a mean split, were too similar in CES-D scores. To investigate this possibility, we repeated the main analyses, using a more extreme group differentiation on the basis of the 33rd (< 8) and 66th (> 18) percentiles of the CES-D scores. Figure D1 provides a comparison of the above 66th percentile and below 33rd percentile groups' performance on the CA and FR task for both dependent variables. Participants with a CES-D score between or equaling the 33rd and 66th percentiles were excluded from these analyses, resulting in a sample of 154 participants. The main analyses were repeated on the resulting, more extreme, experimental groups. For both dependent variables, the ANOVAs (type II) showed a significant main effect of task, $F(1, 150) = 132.47$, $p < 0.001$, $MSe = 46.81$, generalized eta squared (η^2_G) = 0.30, for *total correct* responses, and $F(1, 150) = 160.60$, $p < 0.001$, $MSe = 3.67$, $\eta^2_G = 0.34$, for *blocks-to-learn.* Participants performed better on the CA task, compared to the FR task. Additionally, the interaction between order and task was significant, $F(1, 150) = 7.61$, $p < 0.01$, $MSe = 46.81$, $\eta^2_G = 0.02$, for *total correct*, and $F(1, 150) = 6.31$, $p = 0.01$, $MSe = 3.67$, $\eta^2_G = 0.02$, for *blocks-to-learn.* Participants showed a better performance on the task (CA or FR) that was administered first. Crucially, the interaction of main interest, between group and task, was again not significant, $F(1, 150) = 0.98$, $p = 0.32$, $MSe = 46.81$, for *total correct*, and $F(1, 150) = 2.69$, $p = 0.10$, $MSe = 3.67$, for *blocks-to-learn.*

When the categorical variable distinguishing between a group with high versus low depressive symptoms was replaced by a continuous predictor, the CES-D sum score, again no significant interaction between CESD sum score and task could be observed, $t(234) = -0.31$, $p = 0.75$, for *total correct*, and $t(234) = -1.13$, $p = 0.26$, for *blocks-to-learn*.

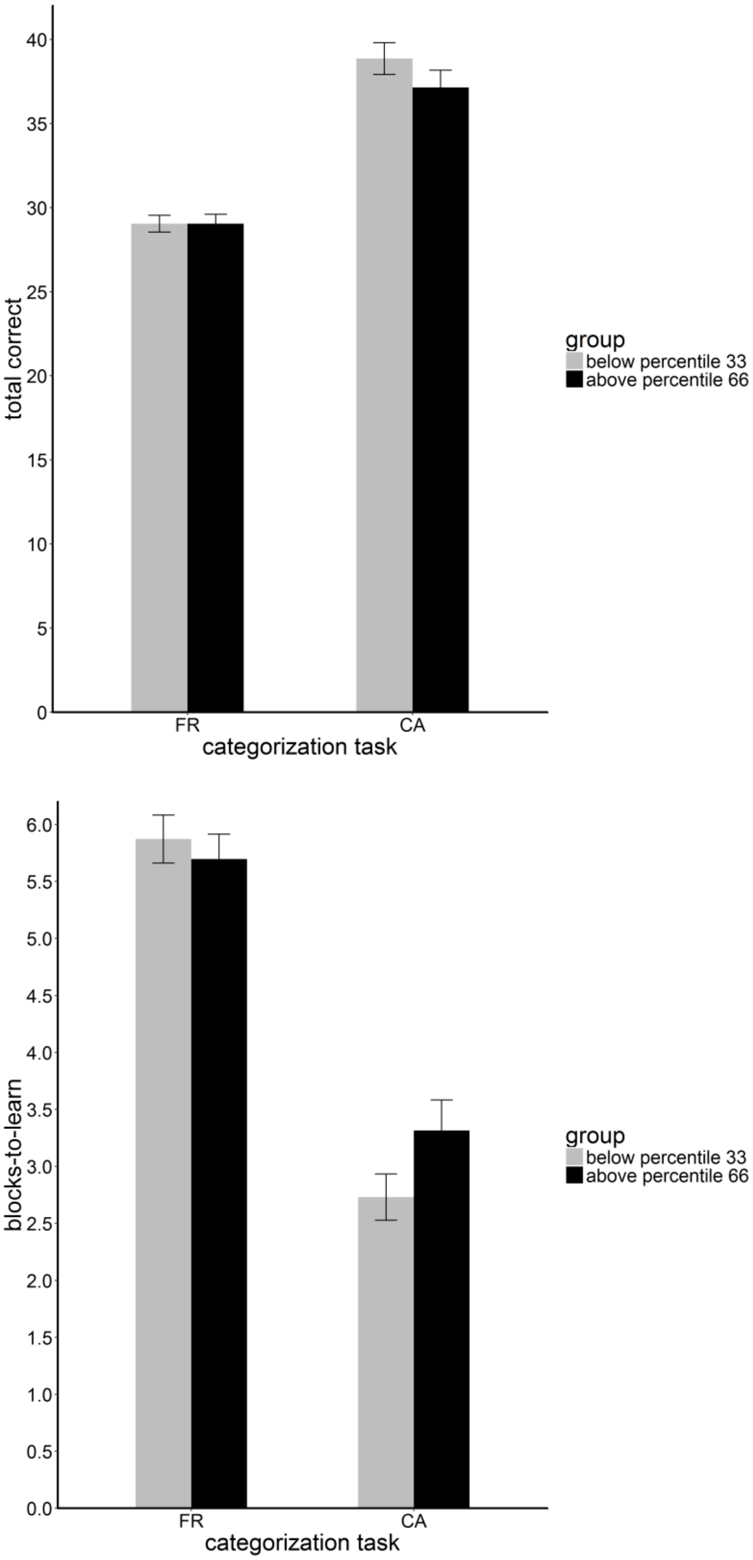

Figure D1. Performance on the FR and CA tasks by people with a CES-D score below the 33rd percentile or above the 66th percentile, with error bars showing the standard errors of the sample means.

References

Pinheiro J., Bates D., DebRoy S., Sarkar D., and R Core Team (2017). nlme: Linear and Nonlinear Mixed Effects Models. R package version 3.1-131,https://CRAN.R-project.org/package=nlme.

Wills, A. J., Inkster, A. B., & Milton, F. (2015). Combination or differentiation? Two theories of processing order in classification. *Cognitive Psychology*, *80*, 1-33.
